# Circulating serum vitamin D levels and total body bone mineral density: A Mendelian randomization study

**DOI:** 10.1111/jcmm.14153

**Published:** 2019-01-13

**Authors:** Jing‐yi Sun, Ming Zhao, Yajun Hou, Cheng Zhang, Jinrok Oh, Zheng Sun, Bao‐liang Sun

**Affiliations:** ^1^ Department of orthopedics Wonju Severance Christian Hospital, Yonsei University Wonju College of Medicine Wonju Gangwon Republic of Korea; ^2^ Department of orthopedics Taian City Central Hospital Taian Shandong P.R. China; ^3^ Affiliated Hospital & Key Laboratory of Cerebral Microcirculation in Universities of Shandong Taishan Medical University Taian Shandong P.R. China; ^4^ Second School of Clinical Medicine Taishan Medical University Taian Shandong P.R. China

**Keywords:** bone mineral density, Mendelian randomization, vitamin D

## Abstract

Until recently, randomized controlled trials have not demonstrated convincing evidence that vitamin D, or vitamin D in combination with calcium supplementation could improve bone mineral density (BMD), osteoporosis and fracture. It remains unclear whether vitamin D levels are causally associated with total body BMD. Here, we performed a Mendelian randomization study to investigate the association of vitamin D levels with total body BMD using a large‐scale vitamin D genome‐wide association study (GWAS) dataset (including 79 366 individuals) and a large‐scale total body BMD GWAS dataset (including 66,628 individuals). We selected three Mendelian randomization methods including inverse‐variance weighted meta‐analysis (IVW), weighted median regression and MR‐Egger regression. All these three methods did not show statistically significant association of genetically increased vitamin D levels with total body BMD. Importantly, our findings are consistent with recent randomized clinical trials and Mendelian randomization study. In summary, we provide genetic evidence that increased vitamin D levels could not improve BMD in the general population. Hence, vitamin D supplementation alone may not be associated with reduced fracture incidence among community‐dwelling adults without known vitamin D deficiency, osteoporosis, or prior fracture.

## 1 INTRODUCTION

Calcium is involved in many biological processes.^1^ Vitamin D plays an important role in skeletal health by regulating calcium and phosphorus metabolism.^2^ It is well known that calcium deficiency could cause osteoporosis.^3^ Osteoporosis is a common systemic skeletal disease characterized by an increased propensity to fracture.^3^ Osteoporosis could be diagnosed mainly through the measurement of bone mineral density (BMD).^3^ Until now, randomized controlled trials have not demonstrated convincing evidence that vitamin D, or vitamin D in combination with calcium supplementation could improve BMD, osteoporosis and fracture.^4^, ^5^, ^6^ Until now, it remains unclear whether vitamin D levels are causally associated with BMD, osteoporosis and fracture. Here, a Mendelian randomization study was performed to investigate the genetic association between vitamin D levels and total body BMD using a large‐scale vitamin D GWAS dataset and a large‐scale total body BMD GWAS dataset.

## 2 MATERIALS AND METHODS

### 2.1 Vitamin D GWAS dataset

We selected six genetic variants associated with circulating 25‐hydroxyvitamin D (25OHD) levels at a genome‐wide significant level (*P* < 5.00E‐08) from a recent large‐scale genome‐wide association study (GWAS) dataset including 79 366 individuals of European descent.^7^ All these six genetic variants were independent and not in linkage disequilibrium. Here, we selected these six genetic variants influencing 25OHD levels as the potential instrumental variables. The summary results including the effect of each genetic variant on 25OHD levels and the standard errors are exacted from the recent study (Table 1).^7^

**Table 1:** Characteristics of six genetic variants with vitamin D levels

SNP	Position	Nearby genes	EA/NEA	EAF	Beta	SE	*P* value
rs3755967	4:72828262	GC	C/T	0.72	0.089	0.0023	4.74E‐343
rs12785878	11:70845097	NADSYN1/DHCR7	T/G	0.75	0.036	0.0022	3.80E‐62
rs10741657	11:14871454	CYP2R1	A/G	0.40	0.031	0.0022	2.05E‐46
rs17216707	20:52165769	CYP24A1	T/C	0.79	0.026	0.0027	8.14E‐23
rs10745742	12:94882660	AMDHD1	T/C	0.40	0.017	0.0022	1.88E‐14
rs8018720	14:38625936	SEC23A	G/C	0.18	0.017	0.0029	4.72E‐09

EA, Effect Allele; EAF, Effect Allele Frequency; NEA, Non‐Effect Allele; SE, standard error; SNP, single‐nucleotide polymorphism. Beta is the regression coefficient based on the vitamin D raising allele (effect allele). Beta > 0 and Beta < 0 means that this effect allele regulates increased and reduced vitamin D levels or bone mineral density, respectively.

### 2.2 BMD GWAS datasets

The BMD GWAS dataset is from the GEnetic Factors for OSteoporosis (GEFOS) Consortium.^8^ The dataset is a total body BMD GWAS including 66 628 individuals from multiple population‐based cohorts across Europe (86%), America (2%) and Australia (14%).^8^ Meanwhile, single GWAS was performed in each of five age strata spanning 15 years including 0‐15 years (n = 11 807), 15‐30 years (n = 4180), 30‐45 years (n = 10 062), 45‐60 years (n = 18 805) and 60 or more years (N = 22 504).^8^

### 2.3 Pleiotropy analysis

In stage 1, we conducted a systematic literature search to explore the potential modifiable risk factors of BMD. We identified some BMD modifiable risk factors including high blood pressure, type 2 diabetes, low body mass index (BMI), smoking, excessive alcohol intake, rheumatoid arthritis, ulcerative colitis, crohns disease or inflammatory bowel disease and education. Meanwhile, lipid levels were not associated with BMD risk. Here, we evaluated the potential pleiotropic association of each 25OHD genetic variant with the potential confounders including type 2 diabetes from DIAbetes Genetics Replication and Meta‐analysis (DIAGRAM) Consortium, obesity including BMI, waist hip ratio, waist hip ratio adjusted for BMI, waist circumference and hip circumference from Genetic Investigation of ANthropometric Traits (GIANT) consortium, systolic blood pressure and diastolic blood pressure from the International Consortium of Blood Pressure (ICBP) consortium, smoking behaviour from the Tobacco and Genetics Consortium (TGC) (cigarettes smoked per day), alcohol drinking (heavy vs light), rheumatoid arthritis, ulcerative colitis, crohns disease or inflammatory bowel disease from International Inflammatory Bowel Disease Genetics Consortium (IIBDGC) and education from Social Science Genetic Association Consortium (SSGAC). The significance threshold for the association of these six vitamin D genetic variants with the potential confounders is a Bonferroni correction *P < *0.05/6 = 0.008 333. In stage 2, we selected MR‐Egger intercept test to evaluate the pleiotropic associations of these six genetic variants with other potential confounders.^9^

### 2.4 Mendelian randomization analysis

Here, we selected the inverse‐variance weighted meta‐analysis (IVW) as the main analysis method, and selected the weighted median regression and MR‐Egger regression as the sensitivity analysis methods.^9^ The odds ratio as well as 95% confidence interval of total body BMD corresponds to per 0.5‐mg/dL increase (about 1 standard deviation) in vitamin D levels. All analyses were conducted using the R package ‘Mendelian Randomization’.^10^ The significance of statistically significant association between vitamin D levels and BMD was at Bonferroni corrected significance *P* < 0.05/6 = 0.00833.

## 3 RESULTS

### 3.1 Association of vitamin D variants with BMD

Here, we extracted the summary statistics for all these six genetic variants in each of these six total body BMD GWAS datasets, respectively. Some of these six genetic variants were significantly associated with BMD at the Bonferroni corrected significance threshold (*P* < 0.05/6 = 0.0083) (Tables S1‐S6).

### 3.2 Pleiotropy analysis

In stage 1, none of these six genetic variants was significantly associated with known confounders at the Bonferroni corrected significance threshold (*P* < 0.05/6 = 0.008 33), as described in Table S7. In stage 2, using these six genetic variants, MR‐Egger intercept test showed no significant intercept (all *P *> 0.05) in each of these six GWAS datasets (Table 2). Hence, our following analysis will be based on these six genetic variants.

**Table 2:** Genetic association between increased vitamin D levels and bone mineral density (BMD)

Dataset	Methods	OR/Intercept	SE	95% confidence interval_lower	*P *value
Total body	Weighted_median	0.88	0.066	[0.78, 1.00]	0.057
Total body	IVW	0.92	0.059	[0.82, 1.04]	0.17
Total body	MR‐Egger	0.84	0.105	[0.68, 1.03]	0.096
Total body	MR‐Egger intercept test	0.005	0.005	[−0.004, 0.014]	0.278
Total body (0‐15)	Weighted_median	0.89	0.153	[0.66, 1.20]	0.432
Total body (0‐15)	IVW	0.90	0.138	[0.69, 1.18]	0.44
Total body (0‐15)	MR‐Egger	0.84	0.245	[0.52, 1.35]	0.465
Total body (0‐15)	MR‐Egger intercept test	0.004	0.011	[−0.017, 0.025]	0.72
Total body (15‐30)	Weighted_median	0.97	0.272	[0.57, 1.65]	0.9
Total body (15‐30)	IVW	0.88	0.242	[0.55, 1.41]	0.595
Total body (15‐30)	MR‐Egger	0.83	0.436	[0.35, 1.95]	0.67
Total body (15‐30)	MR‐Egger intercept test	0.003	0.019	[−0.034, 0.04]	0.875
Total body (30‐45)	Weighted_median	0.75	0.168	[0.54, 1.04]	0.081
Total body (30‐45)	IVW	0.81	0.206	[0.54, 1.21]	0.308
Total body (30‐45)	MR‐Egger	0.80	0.41	[0.36, 1.78]	0.582
Total body (30‐45)	MR‐Egger intercept test	0.001	0.018	[−0.035, 0.037]	0.964
Total body (45‐60)	Weighted_median	0.83	0.129	[0.64, 1.07]	0.145
Total body (45‐60)	IVW	0.88	0.174	[0.63, 1.24]	0.476
Total body (45‐60)	MR‐Egger	0.63	0.278	[0.36, 1.08]	0.094
Total body (45‐60)	MR‐Egger intercept test	0.018	0.012	[−0.006, 0.043]	0.138
Total body (>60)	Weighted_median	1.02	0.113	[0.82, 1.28]	0.855
Total body (>60)	IVW	1.02	0.101	[0.84, 1.24]	0.851
Total body (>60)	MR‐Egger	1.14	0.18	[0.80, 1.62]	0.482
Total body (>60)	MR‐Egger intercept test	−0.006	0.008	[−0.021, 0.01]	0.47

IVW, Inverse‐variance weighted meta‐analysis.

The significance of suggestive association between vitamin D levels and BMD was at *P < *0.05.

The significance of statistically significant association between vitamin D levels and BMD was at Bonferroni corrected significance *P* < 0.05/6 = 0.0083.

### 3.3 Association of vitamin D levels with BMD

In the overall dataset and five age stratum datasets, IVW, weighted median regression and MR‐Egger regression IVW did not show statistically significant association of genetically increased vitamin D levels with total body BMD at the Bonferroni corrected significance *P* < 0.05/6 = 0.008 33, as described in Table 2. The leave‐one‐out permutation analysis further showed that the direction and precision of the genetic estimates between increased vitamin D levels and total body BMD remained largely unchanged.

## 4 DISCUSSION

Here, we performed a Mendelian randomization study, and identified no significant association of genetically increased vitamin D levels with BMD of total body in any age stratum. It is worth mentioning that the vitamin D levels were observed by the population‐based studies including up to 61 079 individuals of European descent.^11^ Therefore, our conclusions reflect the effects of vitamin D levels in the general population. These conclusions may be applicable to non‐institutionalized or community‐dwelling asymptomatic adults without a history of fractures. However, these conclusions may not be applicable to patients with osteoporosis, or a history of fractures, or poor vitamin D intake.

Importantly, our findings are consistent with recent randomized clinical trials and Mendelian randomization study. In 2017, Zhou et al analysed a total of 33 randomized trials involving 51 145 community‐dwelling older adults.^6^ They selected the hip fracture as the primary outcome, and non‐vertebral fracture, vertebral fracture and total fracture as the secondary outcomes.^6^ Zhou et al reported no significant association of calcium, or vitamin D or both supplements with risk of hip fracture compared with placebo or no treatment.^6^ In 2018, the United States Preventive Services Task Force (USPSTF) provided an evidence report, systematic review and recommendation statement about the vitamin D, calcium or combined supplementation for the primary prevention of fractures in community‐dwelling adults.^12^, ^13^ The USPSTF analysed 11 randomized clinical trials (N = 51 419) in adults 50 years and older conducted over 2 to 7 years.^12^, ^13^ The USPSTF found adequate evidence that daily supplementation with 400 IU or less of vitamin D and 1000 mg or less of calcium has no benefit for the primary prevention of fractures in community‐dwelling, post‐menopausal women.^12^, ^13^ In 2018, Larsson et al conducted a Mendelian randomization study, and found no causal association between elevated circulating serum vitamin D levels and BMD of femoral neck, lumbar spine and heel in generally healthy populations.^14^ If elevated vitamin D levels are not causally associated with the BMD in the generally healthy population, then long time vitamin D supplementation could not improve BMD. Therefore, our findings may explain why randomized controlled trials have not achieved convincing evidence that vitamin D supplements could improve BMD.

## CONFLICT OF INTEREST

The authors confirm that there are no conflicts of interest.

## AUTHOR CONTRIBUTIONS

JYS and BLS conceived and initiated the project. JYS analysed the data, drew the figures, and wrote the first draft of the manuscript. All authors contributed to the interpretation of the results and critical revision of the manuscript for important intellectual content and approved the final version of the manuscript.
