# Supplementary material for: Circulating serum vitamin D levels and total body bone mineral density: A Mendelian randomization study
Source: J Cell Mol Med. 2019 Jan 13;23(3):2268–71. doi: 10.1111/jcmm.14153 (PMC6378199; doi:10.1111/jcmm.14153)
Supplement: Supplementary file 1 [file JCMM-23-2268-s001.doc]

**Supplementary Data**

**eMethods**

**eTable 1**. Summary results of 6 vitamin D variants with total body BMD in overall individuals

**eTable 2**. Summary results of 6 vitamin D variants with total body BMD in individuals 0-15 years

**eTable 3**. Summary results of 6 vitamin D variants with total body BMD in individuals 15-30 years

**eTable 4**. Summary results of 6 vitamin D variants with total body BMD in individuals 30-45 years

**eTable 5**. Summary results of 6 vitamin D variants with total body BMD in individuals 45-60 years

**eTable 6**. Summary results of 6 vitamin D variants with total body BMD in individuals 60 or more years

**eTable 7**. Associations of 6 vitamin D variants with potential risk factors

**eMethods**

**Pleiotropy analysis**

In stage 1, we conducted a systematic literature search to explore the potential modifiable risk factors of BMD. We identified some BMD modifiable risk factors including high blood pressure [1], type 2 diabetes [2-3], low body mass index (BMI) [3], smoking [4-6], excessive alcohol intake [7-8], rheumatoid arthritis [9], ulcerative colitis, crohns disease, or inflammatory bowel disease [10-11], and education [12]. Meanwhile, lipid levels were not associated with BMD risk [13]. Here, we evaluated the potential pleiotropic association of each serum calcium-associated genetic variant with the potential confounders including type 2 diabetes from DIAbetes Genetics Replication and Meta-analysis (DIAGRAM) Consortium [14], obesity including body mass index (BMI) [15], waist hip ratio, waist hip ratio adjusted for BMI, waist circumference and hip circumference from Genetic Investigation of ANthropometric Traits (GIANT) consortium [16], systolic blood pressure (SBP) and diastolic blood pressure (DBP) from the International Consortium of Blood Pressure (ICBP) consortium [17], smoking behavior from the Tobacco and Genetics Consortium (TGC) (cigarettes smoked per day) [18], alcohol drinking (heavy vs. light) [19], rheumatoid arthritis [9], ulcerative colitis, crohns disease, or inflammatory bowel disease from International Inflammatory Bowel Disease Genetics Consortium (IIBDGC) [10-11], and education from Social Science Genetic Association Consortium (SSGAC) [12,20]. The significance threshold for the association of these 6 vitamin D genetic variants with the potential confounders is a Bonferroni correction *P* < 0.05/6=0.008333. In stage 2, we selected MR-Egger intercept test to evaluate the pleiotropic associations of these 6 genetic variants with other potential confounders [21].

**eTable 1**. Summary results of 6 vitamin D variants with total body-BMD in all individuals [22]

| SNP | EA | NEA | EAF | Beta | SE | *P* value | N |
| --- | --- | --- | --- | --- | --- | --- | --- |
| rs3755967 | t | c | 0.2711 | 0.0121 | 0.0063 | 0.05593 | 66615 |
| rs12785878 | t | g | 0.6797 | 0.0068 | 0.0064 | 0.2839 | 66171 |
| rs8018720 | c | g | 0.8259 | 0.0047 | 0.0076 | 0.537 | 66115 |
| rs10741657 | a | g | 0.393 | -0.0032 | 0.0058 | 0.5805 | 66160 |
| rs10745742 | t | c | 0.4107 | 0.0026 | 0.0058 | 0.6517 | 66619 |
| rs17216707 | t | c | 0.8035 | 0.0021 | 0.0074 | 0.7804 | 64962 |

SNP, single-nucleotide polymorphism; EA, Effect Allele; NEA, Non-Effect Allele; EAF, Effect Allele Frequency; SE, standard error. Beta > 0 and Beta < 0 means that this effect allele regulates increased and reduced BMD, respectively.

**eTable 2**. Summary results of 6 vitamin D variants with total body-BMD in individuals 0-15 years [22]

| SNP | EA | NEA | EAF | Beta | SE | *P* value | N |
| --- | --- | --- | --- | --- | --- | --- | --- |
| rs10741657 | a | g | 0.385 | -0.0124 | 0.0136 | 0.3596 | 11360 |
| rs3755967 | t | c | 0.2674 | 0.0115 | 0.0148 | 0.4365 | 11807 |
| rs17216707 | t | c | 0.8074 | -0.0123 | 0.0172 | 0.4759 | 11360 |
| rs10745742 | t | c | 0.409 | 0.0079 | 0.013 | 0.5446 | 11807 |
| rs12785878 | t | g | 0.6658 | 0.0075 | 0.0141 | 0.595 | 11360 |
| rs8018720 | c | g | 0.824 | -0.0011 | 0.0172 | 0.9489 | 11360 |

SNP, single-nucleotide polymorphism; EA, Effect Allele; NEA, Non-Effect Allele; EAF, Effect Allele Frequency; SE, standard error. Beta > 0 and Beta < 0 means that this effect allele regulates increased and reduced BMD, respectively.

**eTable 3**. Summary results of 6 vitamin D variants with total body-BMD in individuals 15-30 years [22]

| SNP | EA | NEA | EAF | Beta | SE | *P* value | N |
| --- | --- | --- | --- | --- | --- | --- | --- |
| rs10741657 | a | g | 0.3943 | -0.0301 | 0.0231 | 0.1926 | 4175 |
| rs12785878 | t | g | 0.6617 | -0.0244 | 0.0245 | 0.3189 | 4179 |
| rs8018720 | c | g | 0.8353 | -0.0286 | 0.0307 | 0.3511 | 4175 |
| rs10745742 | t | c | 0.4029 | 0.0198 | 0.0233 | 0.3964 | 4179 |
| rs17216707 | t | c | 0.7978 | -0.0026 | 0.0297 | 0.9293 | 4161 |
| rs3755967 | t | c | 0.2573 | 3.00E-04 | 0.0263 | 0.9915 | 4180 |

SNP, single-nucleotide polymorphism; EA, Effect Allele; NEA, Non-Effect Allele; EAF, Effect Allele Frequency; SE, standard error. Beta > 0 and Beta < 0 means that this effect allele regulates increased and reduced BMD, respectively.

**eTable 4**. Summary results of 6 vitamin D variants with total body-BMD in individuals 30-45 years [22]

| SNP | EA | NEA | EAF | Beta | SE | *P* value | N |
| --- | --- | --- | --- | --- | --- | --- | --- |
| rs10745742 | t | c | 0.419 | -0.0287 | 0.0151 | 0.05777 | 10062 |
| rs3755967 | t | c | 0.2702 | 0.0264 | 0.0161 | 0.1015 | 10060 |
| rs17216707 | t | c | 0.8038 | 0.0309 | 0.0189 | 0.1019 | 10031 |
| rs8018720 | c | g | 0.8196 | 0.0187 | 0.0195 | 0.3386 | 10051 |
| rs10741657 | a | g | 0.4016 | 0.0142 | 0.0149 | 0.3412 | 10055 |
| rs12785878 | t | g | 0.651 | -0.0102 | 0.0165 | 0.5356 | 10062 |

SNP, single-nucleotide polymorphism; EA, Effect Allele; NEA, Non-Effect Allele; EAF, Effect Allele Frequency; SE, standard error. Beta > 0 and Beta < 0 means that this effect allele regulates increased and reduced BMD, respectively.

**eTable 5**. Summary results of 6 vitamin D variants with total body-BMD in individuals 45-60 years [22]

| SNP | EA | NEA | EAF | Beta | SE | *P* value | N |
| --- | --- | --- | --- | --- | --- | --- | --- |
| rs3755967 | t | c | 0.2747 | 0.028 | 0.0118 | 0.01745 | 18802 |
| rs12785878 | t | g | 0.7096 | 0.0234 | 0.0122 | 0.05639 | 18803 |
| rs10745742 | t | c | 0.4052 | 0.0167 | 0.0109 | 0.1238 | 18802 |
| rs8018720 | c | g | 0.8224 | 0.0188 | 0.0142 | 0.1858 | 18777 |
| rs17216707 | t | c | 0.8057 | 0.0055 | 0.0138 | 0.6901 | 18763 |
| rs10741657 | a | g | 0.3972 | 0.0022 | 0.0108 | 0.8375 | 18802 |

SNP, single-nucleotide polymorphism; EA, Effect Allele; NEA, Non-Effect Allele; EAF, Effect Allele Frequency; SE, standard error. Beta > 0 and Beta < 0 means that this effect allele regulates increased and reduced BMD, respectively.

**eTable 6**. Summary results of 6 vitamin D variants with total body-BMD in individuals 60 or more years [22]

| SNP | EA | NEA | EAF | Beta | SE | *P* value | N |
| --- | --- | --- | --- | --- | --- | --- | --- |
| rs12785878 | t | g | 0.6791 | 0.0137 | 0.0109 | 0.2089 | 22496 |
| rs10741657 | a | g | 0.3928 | -0.0078 | 0.0099 | 0.4326 | 22495 |
| rs10745742 | t | c | 0.4141 | -0.0075 | 0.0099 | 0.4514 | 22499 |
| rs8018720 | c | g | 0.8308 | 0.0045 | 0.013 | 0.7294 | 22474 |
| rs3755967 | t | c | 0.2728 | -0.003 | 0.0108 | 0.7849 | 22496 |
| rs17216707 | t | c | 0.8011 | -0.0023 | 0.0128 | 0.8588 | 21355 |

SNP, single-nucleotide polymorphism; EA, Effect Allele; NEA, Non-Effect Allele; EAF, Effect Allele Frequency; SE, standard error. Beta > 0 and Beta < 0 means that this effect allele regulates increased and reduced BMD, respectively.

**eTable 7**. *P* Values for Associations of 6 vitamin D Variants with potential risk factors

| SNP | Pos (hg19) | Alleles | Trait | Study | PMID | Source | P | N |
| --- | --- | --- | --- | --- | --- | --- | --- | --- |
| rs10741657 | chr11:14914878 | A/G | BMI | GIANT | 25673413 | GIANT | 0.02363 | 233943 |
| rs17216707 | chr20:52732362 | C/T | BMI | GIANT | 25673413 | GIANT | 0.403 | 320911 |
| rs3755967 | chr4:72609398 | T/C | BMI | GIANT | 25673413 | GIANT | 0.5203 | 233648 |
| rs12785878 | chr11:71167449 | G/T | BMI | GIANT | 25673413 | GIANT | 0.6857 | 234017 |
| rs10745742 | chr12:96358529 | C/T | BMI | GIANT | 25673413 | GIANT | 0.7522 | 233727 |
| rs8018720 | chr14:39556185 | C/G | BMI | GIANT | 25673413 | GIANT | 0.9687 | 233939 |
| rs8018720 | chr14:39556185 | C/G | Body fat percentage | Lu Y | 26833246 | Lu Y | 0.044 | 75598 |
| rs10741657 | chr11:14914878 | A/G | Body fat percentage | Lu Y | 26833246 | Lu Y | 0.5245 | 74341 |
| rs10745742 | chr12:96358529 | C/T | Body fat percentage | Lu Y | 26833246 | Lu Y | 0.5922 | 74068 |
| rs17216707 | chr20:52732362 | C/T | Body fat percentage | Lu Y | 26833246 | Lu Y | 0.6729 | 99923 |
| rs12785878 | chr11:71167449 | G/T | Body fat percentage | Lu Y | 26833246 | Lu Y | 0.8788 | 76134 |
| rs3755967 | chr4:72609398 | T/C | Body fat percentage | Lu Y | 26833246 | Lu Y | 0.9336 | 76122 |
| rs10745742 | chr12:96358529 | C/T | Cigarettes per day | TAG | 20418890 | TAG | 0.1084 | 38181 |
| rs12785878 | chr11:71167449 | G/T | Cigarettes per day | TAG | 20418890 | TAG | 0.1834 | 38181 |
| rs17216707 | chr20:52732362 | C/T | Cigarettes per day | TAG | 20418890 | TAG | 0.6596 | 38181 |
| rs3755967 | chr4:72609398 | T/C | Cigarettes per day | TAG | 20418890 | TAG | 0.7744 | 38181 |
| rs8018720 | chr14:39556185 | C/G | Cigarettes per day | TAG | 20418890 | TAG | 0.8839 | 38181 |
| rs10741657 | chr11:14914878 | A/G | Cigarettes per day | TAG | 20418890 | TAG | 0.8965 | 38181 |
| rs8018720 | chr14:39556185 | C/G | College completion | SSGAC | 23722424 | SSGAC | 0.1114 | 95427 |
| rs12785878 | chr11:71167449 | G/T | College completion | SSGAC | 23722424 | SSGAC | 0.1543 | 95427 |
| rs10741657 | chr11:14914878 | A/G | College completion | SSGAC | 23722424 | SSGAC | 0.4006 | 95427 |
| rs3755967 | chr4:72609398 | T/C | College completion | SSGAC | 23722424 | SSGAC | 0.5609 | 95427 |
| rs17216707 | chr20:52732362 | C/T | College completion | SSGAC | 23722424 | SSGAC | 0.6568 | 95427 |
| rs10745742 | chr12:96358529 | C/T | College completion | SSGAC | 23722424 | SSGAC | 0.9727 | 95427 |
| rs3755967 | chr4:72609398 | T/C | Continuous (log10 grams/day) alcohol traits | AlcGen and CHARGE + | 27911795 | AlcGen and CHARGE + | 0.1192 | 70,460 |
| rs10745742 | chr12:96358529 | C/T | Continuous (log10 grams/day) alcohol traits | AlcGen and CHARGE + | 27911795 | AlcGen and CHARGE + | 0.1201 | 70,460 |
| rs8018720 | chr14:39556185 | C/G | Continuous (log10 grams/day) alcohol traits | AlcGen and CHARGE + | 27911795 | AlcGen and CHARGE + | 0.206 | 70,460 |
| rs12785878 | chr11:71167449 | G/T | Continuous (log10 grams/day) alcohol traits | AlcGen and CHARGE + | 27911795 | AlcGen and CHARGE + | 0.477 | 70,460 |
| rs10741657 | chr11:14914878 | A/G | Continuous (log10 grams/day) alcohol traits | AlcGen and CHARGE + | 27911795 | AlcGen and CHARGE + | 0.5243 | 70,460 |
| rs17216707 | chr20:52732362 | C/T | Continuous (log10 grams/day) alcohol traits | AlcGen and CHARGE + | 27911795 | AlcGen and CHARGE + | 0.9681 | 70,460 |
| rs8018720 | chr14:39556185 | C/G | Crohns disease | IBDGC | 26192919 | IBDGC | 0.1655 | 20883 |
| rs3755967 | chr4:72609398 | T/C | Crohns disease | IBDGC | 26192919 | IBDGC | 0.5114 | 20883 |
| rs12785878 | chr11:71167449 | G/T | Crohns disease | IBDGC | 26192919 | IBDGC | 0.5121 | 20883 |
| rs10745742 | chr12:96358529 | C/T | Crohns disease | IBDGC | 26192919 | IBDGC | 0.5736 | 20883 |
| rs10741657 | chr11:14914878 | A/G | Crohns disease | IBDGC | 26192919 | IBDGC | 0.5975 | 20883 |
| rs17216707 | chr20:52732362 | C/T | Crohns disease | IBDGC | 26192919 | IBDGC | 0.6321 | 20883 |
| rs8018720 | chr14:39556185 | C/G | DBP | BPExome | 27618447 | BPExome | 0.09641 | 192763 |
| rs17216707 | chr20:52732362 | C/T | DBP | ICBP | 21909115 | ICBP | 0.105 | 69395 |
| rs10745742 | chr12:96358529 | C/T | DBP | ICBP | 21909115 | ICBP | 0.119 | 69395 |
| rs12785878 | chr11:71167449 | G/T | DBP | ICBP | 21909115 | ICBP | 0.121 | 69395 |
| rs10741657 | chr11:14914878 | A/G | DBP | BPExome | 27618447 | BPExome | 0.1876 | 192763 |
| rs10741657 | chr11:14914878 | A/G | DBP | ICBP | 21909115 | ICBP | 0.587 | 69395 |
| rs12785878 | chr11:71167449 | G/T | DBP | BPExome | 27618447 | BPExome | 0.6109 | 192763 |
| rs3755967 | chr4:72609398 | T/C | DBP | ICBP | 21909115 | ICBP | 0.632 | 69395 |
| rs8018720 | chr14:39556185 | C/G | DBP | ICBP | 21909115 | ICBP | 0.642 | 69395 |
| rs17216707 | chr20:52732362 | C/T | Dichotomous (heavy vs light) alcohol traits | AlcGen and CHARGE + | 27911795 | AlcGen and CHARGE + | 0.1564 | 70,460 |
| rs10745742 | chr12:96358529 | C/T | Dichotomous (heavy vs light) alcohol traits | AlcGen and CHARGE + | 27911795 | AlcGen and CHARGE + | 0.2356 | 70,460 |
| rs8018720 | chr14:39556185 | C/G | Dichotomous (heavy vs light) alcohol traits | AlcGen and CHARGE + | 27911795 | AlcGen and CHARGE + | 0.5533 | 70,460 |
| rs3755967 | chr4:72609398 | T/C | Dichotomous (heavy vs light) alcohol traits | AlcGen and CHARGE + | 27911795 | AlcGen and CHARGE + | 0.6827 | 70,460 |
| rs12785878 | chr11:71167449 | G/T | Dichotomous (heavy vs light) alcohol traits | AlcGen and CHARGE + | 27911795 | AlcGen and CHARGE + | 0.848 | 70,460 |
| rs10741657 | chr11:14914878 | A/G | Dichotomous (heavy vs light) alcohol traits | AlcGen and CHARGE + | 27911795 | AlcGen and CHARGE + | 0.9224 | 70,460 |
| rs3755967 | chr4:72609398 | T/C | Ever smoker | TAG | 20418890 | TAG | 0.0596 | 74035 |
| rs17216707 | chr20:52732362 | C/T | Ever smoker | TAG | 20418890 | TAG | 0.3418 | 74035 |
| rs8018720 | chr14:39556185 | C/G | Ever smoker | TAG | 20418890 | TAG | 0.35 | 74035 |
| rs10741657 | chr11:14914878 | A/G | Ever smoker | TAG | 20418890 | TAG | 0.5558 | 74035 |
| rs10745742 | chr12:96358529 | C/T | Ever smoker | TAG | 20418890 | TAG | 0.5749 | 74035 |
| rs12785878 | chr11:71167449 | G/T | Ever smoker | TAG | 20418890 | TAG | 0.628 | 74035 |
| rs10745742 | chr12:96358529 | C/T | Former smoker | TAG | 20418890 | TAG | 0.3037 | 41278 |
| rs12785878 | chr11:71167449 | G/T | Former smoker | TAG | 20418890 | TAG | 0.4734 | 41278 |
| rs3755967 | chr4:72609398 | T/C | Former smoker | TAG | 20418890 | TAG | 0.5288 | 41278 |
| rs17216707 | chr20:52732362 | C/T | Former smoker | TAG | 20418890 | TAG | 0.8879 | 41278 |
| rs10741657 | chr11:14914878 | A/G | Former smoker | TAG | 20418890 | TAG | 0.9061 | 41278 |
| rs8018720 | chr14:39556185 | C/G | Former smoker | TAG | 20418890 | TAG | 0.9237 | 41278 |
| rs10741657 | chr11:14914878 | A/G | Hypertension | BPExome | 27618447 | BPExome | 0.4759 | 183273 |
| rs12785878 | chr11:71167449 | G/T | Hypertension | BPExome | 27618447 | BPExome | 0.6225 | 183273 |
| rs8018720 | chr14:39556185 | C/G | Hypertension | BPExome | 27618447 | BPExome | 0.6281 | 183273 |
| rs10741657 | chr11:14914878 | A/G | Inflammatory bowel disease | IBDGC | 26192919 | IBDGC | 0.2275 | 34652 |
| rs8018720 | chr14:39556185 | C/G | Inflammatory bowel disease | IBDGC | 26192919 | IBDGC | 0.3224 | 34652 |
| rs10745742 | chr12:96358529 | C/T | Inflammatory bowel disease | IBDGC | 26192919 | IBDGC | 0.7349 | 34652 |
| rs12785878 | chr11:71167449 | G/T | Inflammatory bowel disease | IBDGC | 26192919 | IBDGC | 0.7914 | 34652 |
| rs17216707 | chr20:52732362 | C/T | Inflammatory bowel disease | IBDGC | 26192919 | IBDGC | 0.8975 | 34652 |
| rs3755967 | chr4:72609398 | T/C | Inflammatory bowel disease | IBDGC | 26192919 | IBDGC | 0.9668 | 34652 |
| rs8018720 | chr14:39556185 | C/G | Rheumatoid arthritis | Okada Y | 24390342 | Okada Y | 0.017 | 58284 |
| rs3755967 | chr4:72609398 | T/C | Rheumatoid arthritis | Okada Y | 24390342 | Okada Y | 0.56 | 58284 |
| rs12785878 | chr11:71167449 | G/T | Rheumatoid arthritis | Okada Y | 24390342 | Okada Y | 0.65 | 58284 |
| rs10741657 | chr11:14914878 | A/G | Rheumatoid arthritis | Okada Y | 24390342 | Okada Y | 0.84 | 58284 |
| rs10745742 | chr12:96358529 | C/T | Rheumatoid arthritis | Okada Y | 24390342 | Okada Y | 0.88 | 58284 |
| rs17216707 | chr20:52732362 | C/T | Rheumatoid arthritis | Okada Y | 24390342 | Okada Y | 0.97 | 58284 |
| rs17216707 | chr20:52732362 | C/T | SBP | ICBP | 21909115 | ICBP | 0.14 | 69395 |
| rs10745742 | chr12:96358529 | C/T | SBP | ICBP | 21909115 | ICBP | 0.329 | 69395 |
| rs12785878 | chr11:71167449 | G/T | SBP | BPExome | 27618447 | BPExome | 0.4293 | 192763 |
| rs3755967 | chr4:72609398 | T/C | SBP | ICBP | 21909115 | ICBP | 0.467 | 69395 |
| rs10741657 | chr11:14914878 | A/G | SBP | BPExome | 27618447 | BPExome | 0.645 | 192763 |
| rs8018720 | chr14:39556185 | C/G | SBP | ICBP | 21909115 | ICBP | 0.681 | 69395 |
| rs12785878 | chr11:71167449 | G/T | SBP | ICBP | 21909115 | ICBP | 0.703 | 69395 |
| rs8018720 | chr14:39556185 | C/G | SBP | BPExome | 27618447 | BPExome | 0.7141 | 192763 |
| rs10741657 | chr11:14914878 | A/G | SBP | ICBP | 21909115 | ICBP | 0.998 | 69395 |
| rs17216707 | chr20:52732362 | C/T | Type II diabetes | DIAGRAM | 24509480 | DIAGRAM | 0.011 | 110452 |
| rs3755967 | chr4:72609398 | T/C | Type II diabetes | DIAGRAM | 24509480 | DIAGRAM | 0.052 | 110452 |
| rs10745742 | chr12:96358529 | C/T | Type II diabetes | DIAGRAM | 24509480 | DIAGRAM | 0.15 | 110452 |
| rs8018720 | chr14:39556185 | C/G | Type II diabetes | DIAGRAM | 24509480 | DIAGRAM | 0.17 | 110452 |
| rs12785878 | chr11:71167449 | G/T | Type II diabetes | DIAGRAM | 24509480 | DIAGRAM | 0.21 | 110452 |
| rs10741657 | chr11:14914878 | A/G | Type II diabetes | DIAGRAM | 24509480 | DIAGRAM | 0.67 | 110452 |
| rs10741657 | chr11:14914878 | A/G | Ulcerative colitis | IBDGC | 26192919 | IBDGC | 0.1539 | 27432 |
| rs3755967 | chr4:72609398 | T/C | Ulcerative colitis | IBDGC | 26192919 | IBDGC | 0.424 | 27432 |
| rs10745742 | chr12:96358529 | C/T | Ulcerative colitis | IBDGC | 26192919 | IBDGC | 0.5868 | 27432 |
| rs17216707 | chr20:52732362 | C/T | Ulcerative colitis | IBDGC | 26192919 | IBDGC | 0.7037 | 27432 |
| rs8018720 | chr14:39556185 | C/G | Ulcerative colitis | IBDGC | 26192919 | IBDGC | 0.7125 | 27432 |
| rs12785878 | chr11:71167449 | G/T | Ulcerative colitis | IBDGC | 26192919 | IBDGC | 0.8524 | 27432 |
| rs12785878 | chr11:71167449 | G/T | Years of educational attainment | SSGAC | 27225129 | SSGAC | 0.05727 | 328917 |
| rs10745742 | chr12:96358529 | C/T | Years of educational attainment | SSGAC | 27225129 | SSGAC | 0.3216 | 328917 |
| rs10741657 | chr11:14914878 | A/G | Years of educational attainment | SSGAC | 27225129 | SSGAC | 0.429 | 328917 |
| rs3755967 | chr4:72609398 | T/C | Years of educational attainment | SSGAC | 27225129 | SSGAC | 0.5129 | 328917 |
| rs8018720 | chr14:39556185 | C/G | Years of educational attainment | SSGAC | 27225129 | SSGAC | 0.7335 | 328917 |
| rs17216707 | chr20:52732362 | C/T | Years of educational attainment | SSGAC | 27225129 | SSGAC | 0.9082 | 328917 |

**Reference**

1. **Cappuccio FP, Meilahn E, Zmuda JM, Cauley JA.** High blood pressure and bone-mineral loss in elderly white women: a prospective study. Study of Osteoporotic Fractures Research Group. *Lancet*. 1999; 354: 971-5.

2. **Ma L, Oei L, Jiang L, Estrada K, Chen H, Wang Z, Yu Q, Zillikens MC, Gao X, Rivadeneira F.** Association between bone mineral density and type 2 diabetes mellitus: a meta-analysis of observational studies. *Eur J Epidemiol*. 2012; 27: 319-32.

3. **Shanbhogue VV, Mitchell DM, Rosen CJ, Bouxsein ML.** Type 2 diabetes and the skeleton: new insights into sweet bones. *Lancet Diabetes Endocrinol*. 2016; 4: 159-73.

4. **Lorentzon M, Mellstrom D, Haug E, Ohlsson C.** Smoking is associated with lower bone mineral density and reduced cortical thickness in young men. *J Clin Endocrinol Metab*. 2007; 92: 497-503.

5. **Law MR, Hackshaw AK.** A meta-analysis of cigarette smoking, bone mineral density and risk of hip fracture: recognition of a major effect. *BMJ*. 1997; 315: 841-6.

6. **Baron JA, Farahmand BY, Weiderpass E, Michaelsson K, Alberts A, Persson I, Ljunghall S.** Cigarette smoking, alcohol consumption, and risk of hip fracture in women. *Arch Intern Med*. 2001; 161: 983-8.

7. **Jang HD, Hong JY, Han K, Lee JC, Shin BJ, Choi SW, Suh SW, Yang JH, Park SY, Bang C.** Relationship between bone mineral density and alcohol intake: A nationwide health survey analysis of postmenopausal women. *PLoS One*. 2017; 12: e0180132.

8. **McLernon DJ, Powell JJ, Jugdaohsingh R, Macdonald HM.** Do lifestyle choices explain the effect of alcohol on bone mineral density in women around menopause? *Am J Clin Nutr*. 2012; 95: 1261-9.

9. **Lodder MC, de Jong Z, Kostense PJ, Molenaar ET, Staal K, Voskuyl AE, Hazes JM, Dijkmans BA, Lems WF.** Bone mineral density in patients with rheumatoid arthritis: relation between disease severity and low bone mineral density. *Ann Rheum Dis*. 2004; 63: 1576-80.

10. **Scott EM, Gaywood I, Scott BB.** Guidelines for osteoporosis in coeliac disease and inflammatory bowel disease. British Society of Gastroenterology. *Gut*. 2000; 46 Suppl 1: i1-8.

11. **Bjarnason I, Macpherson A, Mackintosh C, Buxton-Thomas M, Forgacs I, Moniz C.** Reduced bone density in patients with inflammatory bowel disease. *Gut*. 1997; 40: 228-33.

12. **Ho SC, Chen YM, Woo JL.** Educational level and osteoporosis risk in postmenopausal Chinese women. *Am J Epidemiol*. 2005; 161: 680-90.

13. **Solomon DH, Avorn J, Canning CF, Wang PS.** Lipid levels and bone mineral density. *Am J Med*. 2005; 118: 1414.

14. **Mahajan A, Go MJ, Zhang W, Below JE, Gaulton KJ, Ferreira T, Horikoshi M, Johnson AD, Ng MC, Prokopenko I, Saleheen D, Wang X, Zeggini E, Abecasis GR, Adair LS, Almgren P, Atalay M, Aung T, Baldassarre D, Balkau B, Bao Y, Barnett AH, Barroso I, Basit A, Been LF, Beilby J, Bell GI, Benediktsson R, Bergman RN, Boehm BO, Boerwinkle E, Bonnycastle LL, Burtt N, Cai Q, Campbell H, Carey J, Cauchi S, Caulfield M, Chan JC, Chang LC, Chang TJ, Chang YC, Charpentier G, Chen CH, Chen H, Chen YT, Chia KS, Chidambaram M, Chines PS, Cho NH, Cho YM, Chuang LM, Collins FS, Cornelis MC, Couper DJ, Crenshaw AT, van Dam RM, Danesh J, Das D, de Faire U, Dedoussis G, Deloukas P, Dimas AS, Dina C, Doney AS, Donnelly PJ, Dorkhan M, van Duijn C, Dupuis J, Edkins S, Elliott P, Emilsson V, Erbel R, Eriksson JG, Escobedo J, Esko T, Eury E, Florez JC, Fontanillas P, Forouhi NG, Forsen T, Fox C, Fraser RM, Frayling TM, Froguel P, Frossard P, Gao Y, Gertow K, Gieger C, Gigante B, Grallert H, Grant GB, Grrop LC, Groves CJ, Grundberg E, Guiducci C, Hamsten A, Han BG, Hara K, Hassanali N, Hattersley AT, Hayward C, Hedman AK, Herder C, Hofman A, Holmen OL, Hovingh K, Hreidarsson AB, Hu C, Hu FB, Hui J, Humphries SE, Hunt SE, Hunter DJ, Hveem K, Hydrie ZI, Ikegami H, Illig T, Ingelsson E, Islam M, Isomaa B, Jackson AU, Jafar T, James A, Jia W, Jockel KH, Jonsson A, Jowett JB, Kadowaki T, Kang HM, Kanoni S, Kao WH, Kathiresan S, Kato N, Katulanda P, Keinanen-Kiukaanniemi KM, Kelly AM, Khan H, Khaw KT, Khor CC, Kim HL, Kim S, Kim YJ, Kinnunen L, Klopp N, Kong A, Korpi-Hyovalti E, Kowlessur S, Kraft P, Kravic J, Kristensen MM, Krithika S, Kumar A, Kumate J, Kuusisto J, Kwak SH, Laakso M, Lagou V, Lakka TA, Langenberg C, Langford C, Lawrence R, Leander K, Lee JM, Lee NR, Li M, Li X, Li Y, Liang J, Liju S, Lim WY, Lind L, Lindgren CM, Lindholm E, Liu CT, Liu JJ, Lobbens S, Long J, Loos RJ, Lu W, Luan J, Lyssenko V, Ma RC, Maeda S, Magi R, Mannisto S, Matthews DR, Meigs JB, Melander O, Metspalu A, Meyer J, Mirza G, Mihailov E, Moebus S, Mohan V, Mohlke KL, Morris AD, Muhleisen TW, Muller-Nurasyid M, Musk B, Nakamura J, Nakashima E, Navarro P, Ng PK, Nica AC, Nilsson PM, Njolstad I, Nothen MM, Ohnaka K, Ong TH, Owen KR, Palmer CN, Pankow JS, Park KS, Parkin M, Pechlivanis S, Pedersen NL, Peltonen L, Perry JR, Peters A, Pinidiyapathirage JM, Platou CG, Potter S, Price JF, Qi L, Radha V, Rallidis L, Rasheed A, Rathman W, Rauramaa R, Raychaudhuri S, Rayner NW, Rees SD, Rehnberg E, Ripatti S, Robertson N, Roden M, Rossin EJ, Rudan I, Rybin D, Saaristo TE, Salomaa V, Saltevo J, Samuel M, Sanghera DK, Saramies J, Scott J, Scott LJ, Scott RA, Segre AV, Sehmi J, Sennblad B, Shah N, Shah S, Shera AS, Shu XO, Shuldiner AR, Sigurdsson G, Sijbrands E, Silveira A, Sim X, Sivapalaratnam S, Small KS, So WY, Stancakova A, Stefansson K, Steinbach G, Steinthorsdottir V, Stirrups K, Strawbridge RJ, Stringham HM, Sun Q, Suo C, Syvanen AC, Takayanagi R, Takeuchi F, Tay WT, Teslovich TM, Thorand B, Thorleifsson G, Thorsteinsdottir U, Tikkanen E, Trakalo J, Tremoli E, Trip MD, Tsai FJ, Tuomi T, Tuomilehto J, Uitterlinden AG, Valladares-Salgado A, Vedantam S, Veglia F, Voight BF, Wang C, Wareham NJ, Wennauer R, Wickremasinghe AR, Wilsgaard T, Wilson JF, Wiltshire S, Winckler W, Wong TY, Wood AR, Wu JY, Wu Y, Yamamoto K, Yamauchi T, Yang M, Yengo L, Yokota M, Young R, Zabaneh D, Zhang F, Zhang R, Zheng W, Zimmet PZ, Altshuler D, Bowden DW, Cho YS, Cox NJ, Cruz M, Hanis CL, Kooner J, Lee JY, Seielstad M, Teo YY, Boehnke M, Parra EJ, Chambers JC, Tai ES, McCarthy MI, Morris AP.** Genome-wide trans-ancestry meta-analysis provides insight into the genetic architecture of type 2 diabetes susceptibility. *Nat Genet*. 2014; 46: 234-44.

15. **Locke AE, Kahali B, Berndt SI, Justice AE, Pers TH, Day FR, Powell C, Vedantam S, Buchkovich ML, Yang J, Croteau-Chonka DC, Esko T, Fall T, Ferreira T, Gustafsson S, Kutalik Z, Luan J, Magi R, Randall JC, Winkler TW, Wood AR, Workalemahu T, Faul JD, Smith JA, Zhao JH, Zhao W, Chen J, Fehrmann R, Hedman AK, Karjalainen J, Schmidt EM, Absher D, Amin N, Anderson D, Beekman M, Bolton JL, Bragg-Gresham JL, Buyske S, Demirkan A, Deng G, Ehret GB, Feenstra B, Feitosa MF, Fischer K, Goel A, Gong J, Jackson AU, Kanoni S, Kleber ME, Kristiansson K, Lim U, Lotay V, Mangino M, Leach IM, Medina-Gomez C, Medland SE, Nalls MA, Palmer CD, Pasko D, Pechlivanis S, Peters MJ, Prokopenko I, Shungin D, Stancakova A, Strawbridge RJ, Sung YJ, Tanaka T, Teumer A, Trompet S, van der Laan SW, van Setten J, Van Vliet-Ostaptchouk JV, Wang Z, Yengo L, Zhang W, Isaacs A, Albrecht E, Arnlov J, Arscott GM, Attwood AP, Bandinelli S, Barrett A, Bas IN, Bellis C, Bennett AJ, Berne C, Blagieva R, Bluher M, Bohringer S, Bonnycastle LL, Bottcher Y, Boyd HA, Bruinenberg M, Caspersen IH, Chen YI, Clarke R, Daw EW, de Craen AJM, Delgado G, Dimitriou M, Doney ASF, Eklund N, Estrada K, Eury E, Folkersen L, Fraser RM, Garcia ME, Geller F, Giedraitis V, Gigante B, Go AS, Golay A, Goodall AH, Gordon SD, Gorski M, Grabe HJ, Grallert H, Grammer TB, Grassler J, Gronberg H, Groves CJ, Gusto G, Haessler J, Hall P, Haller T, Hallmans G, Hartman CA, Hassinen M, Hayward C, Heard-Costa NL, Helmer Q, Hengstenberg C, Holmen O, Hottenga JJ, James AL, Jeff JM, Johansson A, Jolley J, Juliusdottir T, Kinnunen L, Koenig W, Koskenvuo M, Kratzer W, Laitinen J, Lamina C, Leander K, Lee NR, Lichtner P, Lind L, Lindstrom J, Lo KS, Lobbens S, Lorbeer R, Lu Y, Mach F, Magnusson PKE, Mahajan A, McArdle WL, McLachlan S, Menni C, Merger S, Mihailov E, Milani L, Moayyeri A, Monda KL, Morken MA, Mulas A, Muller G, Muller-Nurasyid M, Musk AW, Nagaraja R, Nothen MM, Nolte IM, Pilz S, Rayner NW, Renstrom F, Rettig R, Ried JS, Ripke S, Robertson NR, Rose LM, Sanna S, Scharnagl H, Scholtens S, Schumacher FR, Scott WR, Seufferlein T, Shi J, Smith AV, Smolonska J, Stanton AV, Steinthorsdottir V, Stirrups K, Stringham HM, Sundstrom J, Swertz MA, Swift AJ, Syvanen AC, Tan ST, Tayo BO, Thorand B, Thorleifsson G, Tyrer JP, Uh HW, Vandenput L, Verhulst FC, Vermeulen SH, Verweij N, Vonk JM, Waite LL, Warren HR, Waterworth D, Weedon MN, Wilkens LR, Willenborg C, Wilsgaard T, Wojczynski MK, Wong A, Wright AF, Zhang Q, Brennan EP, Choi M, Dastani Z, Drong AW, Eriksson P, Franco-Cereceda A, Gadin JR, Gharavi AG, Goddard ME, Handsaker RE, Huang J, Karpe F, Kathiresan S, Keildson S, Kiryluk K, Kubo M, Lee JY, Liang L, Lifton RP, Ma B, McCarroll SA, McKnight AJ, Min JL, Moffatt MF, Montgomery GW, Murabito JM, Nicholson G, Nyholt DR, Okada Y, Perry JRB, Dorajoo R, Reinmaa E, Salem RM, Sandholm N, Scott RA, Stolk L, Takahashi A, van 't Hooft FM, Vinkhuyzen AAE, Westra HJ, Zheng W, Zondervan KT, Heath AC, Arveiler D, Bakker SJL, Beilby J, Bergman RN, Blangero J, Bovet P, Campbell H, Caulfield MJ, Cesana G, Chakravarti A, Chasman DI, Chines PS, Collins FS, Crawford DC, Cupples LA, Cusi D, Danesh J, de Faire U, den Ruijter HM, Dominiczak AF, Erbel R, Erdmann J, Eriksson JG, Farrall M, Felix SB, Ferrannini E, Ferrieres J, Ford I, Forouhi NG, Forrester T, Franco OH, Gansevoort RT, Gejman PV, Gieger C, Gottesman O, Gudnason V, Gyllensten U, Hall AS, Harris TB, Hattersley AT, Hicks AA, Hindorff LA, Hingorani AD, Hofman A, Homuth G, Hovingh GK, Humphries SE, Hunt SC, Hypponen E, Illig T, Jacobs KB, Jarvelin MR, Jockel KH, Johansen B, Jousilahti P, Jukema JW, Jula AM, Kaprio J, Kastelein JJP, Keinanen-Kiukaanniemi SM, Kiemeney LA, Knekt P, Kooner JS, Kooperberg C, Kovacs P, Kraja AT, Kumari M, Kuusisto J, Lakka TA, Langenberg C, Marchand LL, Lehtimaki T, Lyssenko V, Mannisto S, Marette A, Matise TC, McKenzie CA, McKnight B, Moll FL, Morris AD, Morris AP, Murray JC, Nelis M, Ohlsson C, Oldehinkel AJ, Ong KK, Madden PAF, Pasterkamp G, Peden JF, Peters A, Postma DS, Pramstaller PP, Price JF, Qi L, Raitakari OT, Rankinen T, Rao DC, Rice TK, Ridker PM, Rioux JD, Ritchie MD, Rudan I, Salomaa V, Samani NJ, Saramies J, Sarzynski MA, Schunkert H, Schwarz PEH, Sever P, Shuldiner AR, Sinisalo J, Stolk RP, Strauch K, Tonjes A, Tregouet DA, Tremblay A, Tremoli E, Virtamo J, Vohl MC, Volker U, Waeber G, Willemsen G, Witteman JC, Zillikens MC, Adair LS, Amouyel P, Asselbergs FW, Assimes TL, Bochud M, Boehm BO, Boerwinkle E, Bornstein SR, Bottinger EP, Bouchard C, Cauchi S, Chambers JC, Chanock SJ, Cooper RS, de Bakker PIW, Dedoussis G, Ferrucci L, Franks PW, Froguel P, Groop LC, Haiman CA, Hamsten A, Hui J, Hunter DJ, Hveem K, Kaplan RC, Kivimaki M, Kuh D, Laakso M, Liu Y, Martin NG, Marz W, Melbye M, Metspalu A, Moebus S, Munroe PB, Njolstad I, Oostra BA, Palmer CNA, Pedersen NL, Perola M, Perusse L, Peters U, Power C, Quertermous T, Rauramaa R, Rivadeneira F, Saaristo TE, Saleheen D, Sattar N, Schadt EE, Schlessinger D, Slagboom PE, Snieder H, Spector TD, Thorsteinsdottir U, Stumvoll M, Tuomilehto J, Uitterlinden AG, Uusitupa M, van der Harst P, Walker M, Wallaschofski H, Wareham NJ, Watkins H, Weir DR, Wichmann HE, Wilson JF, Zanen P, Borecki IB, Deloukas P, Fox CS, Heid IM, O'Connell JR, Strachan DP, Stefansson K, van Duijn CM, Abecasis GR, Franke L, Frayling TM, McCarthy MI, Visscher PM, Scherag A, Willer CJ, Boehnke M, Mohlke KL, Lindgren CM, Beckmann JS, Barroso I, North KE, Ingelsson E, Hirschhorn JN, Loos RJF, Speliotes EK.** Genetic studies of body mass index yield new insights for obesity biology. *Nature*. 2015; 518: 197-206.

16. **Shungin D, Winkler TW, Croteau-Chonka DC, Ferreira T, Locke AE, Magi R, Strawbridge RJ, Pers TH, Fischer K, Justice AE, Workalemahu T, Wu JMW, Buchkovich ML, Heard-Costa NL, Roman TS, Drong AW, Song C, Gustafsson S, Day FR, Esko T, Fall T, Kutalik Z, Luan J, Randall JC, Scherag A, Vedantam S, Wood AR, Chen J, Fehrmann R, Karjalainen J, Kahali B, Liu CT, Schmidt EM, Absher D, Amin N, Anderson D, Beekman M, Bragg-Gresham JL, Buyske S, Demirkan A, Ehret GB, Feitosa MF, Goel A, Jackson AU, Johnson T, Kleber ME, Kristiansson K, Mangino M, Leach IM, Medina-Gomez C, Palmer CD, Pasko D, Pechlivanis S, Peters MJ, Prokopenko I, Stancakova A, Sung YJ, Tanaka T, Teumer A, Van Vliet-Ostaptchouk JV, Yengo L, Zhang W, Albrecht E, Arnlov J, Arscott GM, Bandinelli S, Barrett A, Bellis C, Bennett AJ, Berne C, Bluher M, Bohringer S, Bonnet F, Bottcher Y, Bruinenberg M, Carba DB, Caspersen IH, Clarke R, Daw EW, Deelen J, Deelman E, Delgado G, Doney AS, Eklund N, Erdos MR, Estrada K, Eury E, Friedrich N, Garcia ME, Giedraitis V, Gigante B, Go AS, Golay A, Grallert H, Grammer TB, Grassler J, Grewal J, Groves CJ, Haller T, Hallmans G, Hartman CA, Hassinen M, Hayward C, Heikkila K, Herzig KH, Helmer Q, Hillege HL, Holmen O, Hunt SC, Isaacs A, Ittermann T, James AL, Johansson I, Juliusdottir T, Kalafati IP, Kinnunen L, Koenig W, Kooner IK, Kratzer W, Lamina C, Leander K, Lee NR, Lichtner P, Lind L, Lindstrom J, Lobbens S, Lorentzon M, Mach F, Magnusson PK, Mahajan A, McArdle WL, Menni C, Merger S, Mihailov E, Milani L, Mills R, Moayyeri A, Monda KL, Mooijaart SP, Muhleisen TW, Mulas A, Muller G, Muller-Nurasyid M, Nagaraja R, Nalls MA, Narisu N, Glorioso N, Nolte IM, Olden M, Rayner NW, Renstrom F, Ried JS, Robertson NR, Rose LM, Sanna S, Scharnagl H, Scholtens S, Sennblad B, Seufferlein T, Sitlani CM, Smith AV, Stirrups K, Stringham HM, Sundstrom J, Swertz MA, Swift AJ, Syvanen AC, Tayo BO, Thorand B, Thorleifsson G, Tomaschitz A, Troffa C, van Oort FV, Verweij N, Vonk JM, Waite LL, Wennauer R, Wilsgaard T, Wojczynski MK, Wong A, Zhang Q, Zhao JH, Brennan EP, Choi M, Eriksson P, Folkersen L, Franco-Cereceda A, Gharavi AG, Hedman AK, Hivert MF, Huang J, Kanoni S, Karpe F, Keildson S, Kiryluk K, Liang L, Lifton RP, Ma B, McKnight AJ, McPherson R, Metspalu A, Min JL, Moffatt MF, Montgomery GW, Murabito JM, Nicholson G, Nyholt DR, Olsson C, Perry JR, Reinmaa E, Salem RM, Sandholm N, Schadt EE, Scott RA, Stolk L, Vallejo EE, Westra HJ, Zondervan KT, Amouyel P, Arveiler D, Bakker SJ, Beilby J, Bergman RN, Blangero J, Brown MJ, Burnier M, Campbell H, Chakravarti A, Chines PS, Claudi-Boehm S, Collins FS, Crawford DC, Danesh J, de Faire U, de Geus EJ, Dorr M, Erbel R, Eriksson JG, Farrall M, Ferrannini E, Ferrieres J, Forouhi NG, Forrester T, Franco OH, Gansevoort RT, Gieger C, Gudnason V, Haiman CA, Harris TB, Hattersley AT, Heliovaara M, Hicks AA, Hingorani AD, Hoffmann W, Hofman A, Homuth G, Humphries SE, Hypponen E, Illig T, Jarvelin MR, Johansen B, Jousilahti P, Jula AM, Kaprio J, Kee F, Keinanen-Kiukaanniemi SM, Kooner JS, Kooperberg C, Kovacs P, Kraja AT, Kumari M, Kuulasmaa K, Kuusisto J, Lakka TA, Langenberg C, Le Marchand L, Lehtimaki T, Lyssenko V, Mannisto S, Marette A, Matise TC, McKenzie CA, McKnight B, Musk AW, Mohlenkamp S, Morris AD, Nelis M, Ohlsson C, Oldehinkel AJ, Ong KK, Palmer LJ, Penninx BW, Peters A, Pramstaller PP, Raitakari OT, Rankinen T, Rao DC, Rice TK, Ridker PM, Ritchie MD, Rudan I, Salomaa V, Samani NJ, Saramies J, Sarzynski MA, Schwarz PE, Shuldiner AR, Staessen JA, Steinthorsdottir V, Stolk RP, Strauch K, Tonjes A, Tremblay A, Tremoli E, Vohl MC, Volker U, Vollenweider P, Wilson JF, Witteman JC, Adair LS, Bochud M, Boehm BO, Bornstein SR, Bouchard C, Cauchi S, Caulfield MJ, Chambers JC, Chasman DI, Cooper RS, Dedoussis G, Ferrucci L, Froguel P, Grabe HJ, Hamsten A, Hui J, Hveem K, Jockel KH, Kivimaki M, Kuh D, Laakso M, Liu Y, Marz W, Munroe PB, Njolstad I, Oostra BA, Palmer CN, Pedersen NL, Perola M, Perusse L, Peters U, Power C, Quertermous T, Rauramaa R, Rivadeneira F, Saaristo TE, Saleheen D, Sinisalo J, Slagboom PE, Snieder H, Spector TD, Stefansson K, Stumvoll M, Tuomilehto J, Uitterlinden AG, Uusitupa M, van der Harst P, Veronesi G, Walker M, Wareham NJ, Watkins H, Wichmann HE, Abecasis GR, Assimes TL, Berndt SI, Boehnke M, Borecki IB, Deloukas P, Franke L, Frayling TM, Groop LC, Hunter DJ, Kaplan RC, O'Connell JR, Qi L, Schlessinger D, Strachan DP, Thorsteinsdottir U, van Duijn CM, Willer CJ, Visscher PM, Yang J, Hirschhorn JN, Zillikens MC, McCarthy MI, Speliotes EK, North KE, Fox CS, Barroso I, Franks PW, Ingelsson E, Heid IM, Loos RJ, Cupples LA, Morris AP, Lindgren CM, Mohlke KL.** New genetic loci link adipose and insulin biology to body fat distribution. *Nature*. 2015; 518: 187-96.

17. **Ehret GB, Munroe PB, Rice KM, Bochud M, Johnson AD, Chasman DI, Smith AV, Tobin MD, Verwoert GC, Hwang SJ, Pihur V, Vollenweider P, O'Reilly PF, Amin N, Bragg-Gresham JL, Teumer A, Glazer NL, Launer L, Zhao JH, Aulchenko Y, Heath S, Sober S, Parsa A, Luan J, Arora P, Dehghan A, Zhang F, Lucas G, Hicks AA, Jackson AU, Peden JF, Tanaka T, Wild SH, Rudan I, Igl W, Milaneschi Y, Parker AN, Fava C, Chambers JC, Fox ER, Kumari M, Go MJ, van der Harst P, Kao WH, Sjogren M, Vinay DG, Alexander M, Tabara Y, Shaw-Hawkins S, Whincup PH, Liu Y, Shi G, Kuusisto J, Tayo B, Seielstad M, Sim X, Nguyen KD, Lehtimaki T, Matullo G, Wu Y, Gaunt TR, Onland-Moret NC, Cooper MN, Platou CG, Org E, Hardy R, Dahgam S, Palmen J, Vitart V, Braund PS, Kuznetsova T, Uiterwaal CS, Adeyemo A, Palmas W, Campbell H, Ludwig B, Tomaszewski M, Tzoulaki I, Palmer ND, Aspelund T, Garcia M, Chang YP, O'Connell JR, Steinle NI, Grobbee DE, Arking DE, Kardia SL, Morrison AC, Hernandez D, Najjar S, McArdle WL, Hadley D, Brown MJ, Connell JM, Hingorani AD, Day IN, Lawlor DA, Beilby JP, Lawrence RW, Clarke R, Hopewell JC, Ongen H, Dreisbach AW, Li Y, Young JH, Bis JC, Kahonen M, Viikari J, Adair LS, Lee NR, Chen MH, Olden M, Pattaro C, Bolton JA, Kottgen A, Bergmann S, Mooser V, Chaturvedi N, Frayling TM, Islam M, Jafar TH, Erdmann J, Kulkarni SR, Bornstein SR, Grassler J, Groop L, Voight BF, Kettunen J, Howard P, Taylor A, Guarrera S, Ricceri F, Emilsson V, Plump A, Barroso I, Khaw KT, Weder AB, Hunt SC, Sun YV, Bergman RN, Collins FS, Bonnycastle LL, Scott LJ, Stringham HM, Peltonen L, Perola M, Vartiainen E, Brand SM, Staessen JA, Wang TJ, Burton PR, Soler Artigas M, Dong Y, Snieder H, Wang X, Zhu H, Lohman KK, Rudock ME, Heckbert SR, Smith NL, Wiggins KL, Doumatey A, Shriner D, Veldre G, Viigimaa M, Kinra S, Prabhakaran D, Tripathy V, Langefeld CD, Rosengren A, Thelle DS, Corsi AM, Singleton A, Forrester T, Hilton G, McKenzie CA, Salako T, Iwai N, Kita Y, Ogihara T, Ohkubo T, Okamura T, Ueshima H, Umemura S, Eyheramendy S, Meitinger T, Wichmann HE, Cho YS, Kim HL, Lee JY, Scott J, Sehmi JS, Zhang W, Hedblad B, Nilsson P, Smith GD, Wong A, Narisu N, Stancakova A, Raffel LJ, Yao J, Kathiresan S, O'Donnell CJ, Schwartz SM, Ikram MA, Longstreth WT, Jr., Mosley TH, Seshadri S, Shrine NR, Wain LV, Morken MA, Swift AJ, Laitinen J, Prokopenko I, Zitting P, Cooper JA, Humphries SE, Danesh J, Rasheed A, Goel A, Hamsten A, Watkins H, Bakker SJ, van Gilst WH, Janipalli CS, Mani KR, Yajnik CS, Hofman A, Mattace-Raso FU, Oostra BA, Demirkan A, Isaacs A, Rivadeneira F, Lakatta EG, Orru M, Scuteri A, Ala-Korpela M, Kangas AJ, Lyytikainen LP, Soininen P, Tukiainen T, Wurtz P, Ong RT, Dorr M, Kroemer HK, Volker U, Volzke H, Galan P, Hercberg S, Lathrop M, Zelenika D, Deloukas P, Mangino M, Spector TD, Zhai G, Meschia JF, Nalls MA, Sharma P, Terzic J, Kumar MV, Denniff M, Zukowska-Szczechowska E, Wagenknecht LE, Fowkes FG, Charchar FJ, Schwarz PE, Hayward C, Guo X, Rotimi C, Bots ML, Brand E, Samani NJ, Polasek O, Talmud PJ, Nyberg F, Kuh D, Laan M, Hveem K, Palmer LJ, van der Schouw YT, Casas JP, Mohlke KL, Vineis P, Raitakari O, Ganesh SK, Wong TY, Tai ES, Cooper RS, Laakso M, Rao DC, Harris TB, Morris RW, Dominiczak AF, Kivimaki M, Marmot MG, Miki T, Saleheen D, Chandak GR, Coresh J, Navis G, Salomaa V, Han BG, Zhu X, Kooner JS, Melander O, Ridker PM, Bandinelli S, Gyllensten UB, Wright AF, Wilson JF, Ferrucci L, Farrall M, Tuomilehto J, Pramstaller PP, Elosua R, Soranzo N, Sijbrands EJ, Altshuler D, Loos RJ, Shuldiner AR, Gieger C, Meneton P, Uitterlinden AG, Wareham NJ, Gudnason V, Rotter JI, Rettig R, Uda M, Strachan DP, Witteman JC, Hartikainen AL, Beckmann JS, Boerwinkle E, Vasan RS, Boehnke M, Larson MG, Jarvelin MR, Psaty BM, Abecasis GR, Chakravarti A, Elliott P, van Duijn CM, Newton-Cheh C, Levy D, Caulfield MJ, Johnson T.** Genetic variants in novel pathways influence blood pressure and cardiovascular disease risk. *Nature*. 2011; 478: 103-9.

18. Genome-wide meta-analyses identify multiple loci associated with smoking behavior. *Nat Genet*. 2010; 42: 441-7.

19. **Schumann G, Liu C, O'Reilly P, Gao H, Song P, Xu B, Ruggeri B, Amin N, Jia T, Preis S, Segura Lepe M, Akira S, Barbieri C, Baumeister S, Cauchi S, Clarke TK, Enroth S, Fischer K, Hallfors J, Harris SE, Hieber S, Hofer E, Hottenga JJ, Johansson A, Joshi PK, Kaartinen N, Laitinen J, Lemaitre R, Loukola A, Luan J, Lyytikainen LP, Mangino M, Manichaikul A, Mbarek H, Milaneschi Y, Moayyeri A, Mukamal K, Nelson C, Nettleton J, Partinen E, Rawal R, Robino A, Rose L, Sala C, Satoh T, Schmidt R, Schraut K, Scott R, Smith AV, Starr JM, Teumer A, Trompet S, Uitterlinden AG, Venturini C, Vergnaud AC, Verweij N, Vitart V, Vuckovic D, Wedenoja J, Yengo L, Yu B, Zhang W, Zhao JH, Boomsma DI, Chambers J, Chasman DI, Daniela T, de Geus E, Deary I, Eriksson JG, Esko T, Eulenburg V, Franco OH, Froguel P, Gieger C, Grabe HJ, Gudnason V, Gyllensten U, Harris TB, Hartikainen AL, Heath AC, Hocking L, Hofman A, Huth C, Jarvelin MR, Jukema JW, Kaprio J, Kooner JS, Kutalik Z, Lahti J, Langenberg C, Lehtimaki T, Liu Y, Madden PA, Martin N, Morrison A, Penninx B, Pirastu N, Psaty B, Raitakari O, Ridker P, Rose R, Rotter JI, Samani NJ, Schmidt H, Spector TD, Stott D, Strachan D, Tzoulaki I, van der Harst P, van Duijn CM, Marques-Vidal P, Vollenweider P, Wareham NJ, Whitfield JB, Wilson J, Wolffenbuttel B, Bakalkin G, Evangelou E, Rice KM, Desrivieres S, Kliewer SA, Mangelsdorf DJ, Muller CP, Levy D, Elliott P.** KLB is associated with alcohol drinking, and its gene product beta-Klotho is necessary for FGF21 regulation of alcohol preference. *Proc Natl Acad Sci U S A*. 2016; 113: 14372-7.

20. **Okbay A, Beauchamp JP, Fontana MA, Lee JJ, Pers TH, Rietveld CA, Turley P, Chen GB, Emilsson V, Meddens SF, Oskarsson S, Pickrell JK, Thom K, Timshel P, de Vlaming R, Abdellaoui A, Ahluwalia TS, Bacelis J, Baumbach C, Bjornsdottir G, Brandsma JH, Pina Concas M, Derringer J, Furlotte NA, Galesloot TE, Girotto G, Gupta R, Hall LM, Harris SE, Hofer E, Horikoshi M, Huffman JE, Kaasik K, Kalafati IP, Karlsson R, Kong A, Lahti J, van der Lee SJ, deLeeuw C, Lind PA, Lindgren KO, Liu T, Mangino M, Marten J, Mihailov E, Miller MB, van der Most PJ, Oldmeadow C, Payton A, Pervjakova N, Peyrot WJ, Qian Y, Raitakari O, Rueedi R, Salvi E, Schmidt B, Schraut KE, Shi J, Smith AV, Poot RA, St Pourcain B, Teumer A, Thorleifsson G, Verweij N, Vuckovic D, Wellmann J, Westra HJ, Yang J, Zhao W, Zhu Z, Alizadeh BZ, Amin N, Bakshi A, Baumeister SE, Biino G, Bonnelykke K, Boyle PA, Campbell H, Cappuccio FP, Davies G, De Neve JE, Deloukas P, Demuth I, Ding J, Eibich P, Eisele L, Eklund N, Evans DM, Faul JD, Feitosa MF, Forstner AJ, Gandin I, Gunnarsson B, Halldorsson BV, Harris TB, Heath AC, Hocking LJ, Holliday EG, Homuth G, Horan MA, Hottenga JJ, de Jager PL, Joshi PK, Jugessur A, Kaakinen MA, Kahonen M, Kanoni S, Keltigangas-Jarvinen L, Kiemeney LA, Kolcic I, Koskinen S, Kraja AT, Kroh M, Kutalik Z, Latvala A, Launer LJ, Lebreton MP, Levinson DF, Lichtenstein P, Lichtner P, Liewald DC, Loukola A, Madden PA, Magi R, Maki-Opas T, Marioni RE, Marques-Vidal P, Meddens GA, McMahon G, Meisinger C, Meitinger T, Milaneschi Y, Milani L, Montgomery GW, Myhre R, Nelson CP, Nyholt DR, Ollier WE, Palotie A, Paternoster L, Pedersen NL, Petrovic KE, Porteous DJ, Raikkonen K, Ring SM, Robino A, Rostapshova O, Rudan I, Rustichini A, Salomaa V, Sanders AR, Sarin AP, Schmidt H, Scott RJ, Smith BH, Smith JA, Staessen JA, Steinhagen-Thiessen E, Strauch K, Terracciano A, Tobin MD, Ulivi S, Vaccargiu S, Quaye L, van Rooij FJ, Venturini C, Vinkhuyzen AA, Volker U, Volzke H, Vonk JM, Vozzi D, Waage J, Ware EB, Willemsen G, Attia JR, Bennett DA, Berger K, Bertram L, Bisgaard H, Boomsma DI, Borecki IB, Bultmann U, Chabris CF, Cucca F, Cusi D, Deary IJ, Dedoussis GV, van Duijn CM, Eriksson JG, Franke B, Franke L, Gasparini P, Gejman PV, Gieger C, Grabe HJ, Gratten J, Groenen PJ, Gudnason V, van der Harst P, Hayward C, Hinds DA, Hoffmann W, Hypponen E, Iacono WG, Jacobsson B, Jarvelin MR, Jockel KH, Kaprio J, Kardia SL, Lehtimaki T, Lehrer SF, Magnusson PK, Martin NG, McGue M, Metspalu A, Pendleton N, Penninx BW, Perola M, Pirastu N, Pirastu M, Polasek O, Posthuma D, Power C, Province MA, Samani NJ, Schlessinger D, Schmidt R, Sorensen TI, Spector TD, Stefansson K, Thorsteinsdottir U, Thurik AR, Timpson NJ, Tiemeier H, Tung JY, Uitterlinden AG, Vitart V, Vollenweider P, Weir DR, Wilson JF, Wright AF, Conley DC, Krueger RF, Davey Smith G, Hofman A, Laibson DI, Medland SE, Meyer MN, Johannesson M, Visscher PM, Esko T, Koellinger PD, Cesarini D, Benjamin DJ.** Genome-wide association study identifies 74 loci associated with educational attainment. *Nature*. 2016; 533: 539-42.

21. **Liu G, Zhao Y, Jin S, Hu Y, Wang T, Tian R, Han Z, Xu D, Jiang Q.** Circulating vitamin E levels and Alzheimer's disease: a Mendelian randomization study. *Neurobiol Aging*. 2018; 72: 189 e1- e9.

22. **Medina-Gomez C, Kemp JP, Trajanoska K, Luan J, Chesi A, Ahluwalia TS, Mook-Kanamori DO, Ham A, Hartwig FP, Evans DS, Joro R, Nedeljkovic I, Zheng HF, Zhu K, Atalay M, Liu CT, Nethander M, Broer L, Porleifsson G, Mullin BH, Handelman SK, Nalls MA, Jessen LE, Heppe DHM, Richards JB, Wang C, Chawes B, Schraut KE, Amin N, Wareham N, Karasik D, Van der Velde N, Ikram MA, Zemel BS, Zhou Y, Carlsson CJ, Liu Y, McGuigan FE, Boer CG, Bonnelykke K, Ralston SH, Robbins JA, Walsh JP, Zillikens MC, Langenberg C, Li-Gao R, Williams FMK, Harris TB, Akesson K, Jackson RD, Sigurdsson G, den Heijer M, van der Eerden BCJ, van de Peppel J, Spector TD, Pennell C, Horta BL, Felix JF, Zhao JH, Wilson SG, de Mutsert R, Bisgaard H, Styrkarsdottir U, Jaddoe VW, Orwoll E, Lakka TA, Scott R, Grant SFA, Lorentzon M, van Duijn CM, Wilson JF, Stefansson K, Psaty BM, Kiel DP, Ohlsson C, Ntzani E, van Wijnen AJ, Forgetta V, Ghanbari M, Logan JG, Williams GR, Bassett JHD, Croucher PI, Evangelou E, Uitterlinden AG, Ackert-Bicknell CL, Tobias JH, Evans DM, Rivadeneira F.** Life-Course Genome-wide Association Study Meta-analysis of Total Body BMD and Assessment of Age-Specific Effects. *Am J Hum Genet*. 2018; 102: 88-102.
